# Paradigmatic status of an endo- and exoglucanase and its effect on crystalline cellulose degradation

**DOI:** 10.1186/1754-6834-5-78

**Published:** 2012-10-24

**Authors:** Sarah Moraïs, Yoav Barak, Raphael Lamed, David B Wilson, Qi Xu, Michael E Himmel, Edward A Bayer

**Affiliations:** 1 Department of Biological Chemistry, The Weizmann Institute of Science, Rehovot, Israel; 2 Faculty of Agricultural, Food and Environmental Quality Sciences, The Hebrew University of Jerusalem, P.O. Box 12, Rehovot, 76100, Israel; 3 Chemical Research Support, The Weizmann Institute of Science, Rehovot, 76100, Israel; 4 Department of Molecular Microbiology and Biotechnology, Tel Aviv University, Ramat Aviv, 69978, Israel; 5 Department of Molecular Biology and Genetics, Cornell University, Ithaca, NY, 14853, USA; 6 Biosciences Center, National Renewable Energy Laboratory (NREL) and BioEnergy Science Center (BESC), Golden, CO, USA

**Keywords:** Bifunctional cellulase, *Thermobifida fusca*, Enzyme paradigm

## Abstract

**Background:**

Microorganisms employ a multiplicity of enzymes to efficiently degrade the composite structure of plant cell wall cellulosic polysaccharides. These remarkable enzyme systems include glycoside hydrolases (cellulases, hemicellulases), polysaccharide lyases, and the carbohydrate esterases. To accomplish this challenging task, several strategies are commonly observed either separately or in combination. These include free enzyme systems, multifunctional enzymes, and multi-enzyme self-assembled designer cellulosome complexes.

**Results:**

In order to compare these different paradigms, we employed a synthetic biology approach to convert two different cellulases from the free enzymatic system of the well-studied bacterium, *Thermobifida fusca*, into bifunctional enzymes with different modular architectures. We then examined their performance compared to those of the combined parental free-enzyme and equivalent designer-cellulosome systems. The results showed that the cellulolytic activity displayed by the different architectures of the bifunctional enzymes was somewhat inferior to that of the wild-type free enzyme system.

**Conclusions:**

The activity exhibited by the designer cellulosome system was equal or superior to that of the free system, presumably reflecting the combined proximity of the enzymes and high flexibility of the designer cellulosome components, thus enabling efficient enzymatic activity of the catalytic modules.

## Background

In nature, three dominant microbial paradigms for enzymatic deconstruction of plant cell walls have been observed [1]. Free enzymes, multifunctional enzymes and multi-enzyme complexes (cellulosomes) are common configurations of microbial cellulase systems.

Glycoside hydrolases have been classified thus far in 130 different families [2], which commonly contain a catalytic module that cleaves the glycoside bond and (frequently) a carbohydrate-binding module (CBM) that targets the enzyme to the polysaccharide substrate, and, in many cases, additional types of ancillary modules. Cellulases include endo- and exo-acting enzymes and β-D-glucosidases, which work synergistically to hydrolyze the recalcitrant crystalline cellulose microfibrils of the plant cell wall.

Cellulosomes were discovered in 1983 [3-5] and are composed of non-catalytic scaffoldins, which contain a carbohydrate-binding module (CBM) for substrate targeting, as well as multiple cohesin modules for integrating dockerin-bearing enzymatic subunits to form a multi-component complex. The intermodular cohesin-dockerin interaction dictates the assembly of the cellulosome complex. It is believed that synergistic action is achieved by a combination of substrate-targeting and proximity effects whereby the set of cellulosomal enzymes is collectively concentrated at defined sites on the cellulosic substrate.

A third enzyme paradigm has also emerged: multifunctional enzymes are composed of two or more catalytic modules important for the degradation of plant cell walls [6-11]. They generally contain one or several CBMs and are thus very high-molecular-weight proteins. Dockerin-bearing multifunctional enzymes may also be incorporated into cellulosomes. The presence of several catalytic modules in the same polypeptide chain would seem to indicate that their enforced proximity would account for an enhanced concerted action on cellulosic substrates. To date, four different types of multifunctional enzymes have been described: cellulase-cellulase, hemicellulase-hemicellulase, hemicellulase-cellulase and hemicellulase-carbohydrate esterase systems [1]. Cellulase-cellulase multifunctional enzymes observed to date present a variety of modular architectures and include combination of two catalytic modules (GH6 with GH12, GH9 with GH48, GH5 with GH12 or GH5 with GH6) with a minimum of one CBM (from families 2, 3, 5 and 10) and could contain supplementary ancillary modules such as FN3-like modules [1,12].

The benefits of each strategy have been the subject of many research projects in the field, but remain as yet unclear. The comparison between free enzymatic and cellulosomal systems has been addressed in a number of recent studies [13-23], and it appears that the cellulosomal paradigm offers advantages in deconstructing insoluble cellulosic substrates relative to free enzyme systems.

Another interesting attempt to increase enzyme synergism and compare enzyme paradigms was reported recently in the form of multifunctional enzyme conjugates [24,25]. These authors observed an increase in degradation of natural substrates, upon fusing two or three complementary xylan-degrading activities (xylanase, arabinofuranosidase and xylosidase) into the same polypeptide chain. Other multifunctional enzyme chimeras have been created successfully and revealed promising combined activities [26-28].

Nevertheless, comparison between the three major enzyme paradigms has not been reported in the literature. In this communication, we describe the use of the well-characterized cellulolytic system of *Thermobifida fusca* as a model to compare the performance of the three enzyme paradigms. *T. fusca* possesses a limited set of only six cellulases and does not produce multifunctional enzymes. It thus represents an excellent system to prepare a variety of free and complexed cellulase systems, using the catalytic modules derived from this bacterium. In previous studies [16,18,19,21,22], we have employed synthetic biology approaches to compare both free *T. fusca* enzymes and their respective engineered designer cellulosomes. One advantage of the *T. fusca* system is that even though its enzymes are not cellulosomal, a dockerin module can be readily grafted onto the parent enzyme by replacing the native CBM; the chimaeric enzyme can then be assembled into a complementary scaffoldin to form a designer cellulosome.

In the present study, we compared the combined synergistic action of two *T. fusca* cellulases: (i) in their free state (the wild-type system), (ii) as bifunctional enzymes in a single polypeptide chain (i.e., to mimic the natural multifunctional cellulase-cellulase system) and (iii) as dockerin-bearing enzymes attached to a scaffoldin in the cellulosomal mode. In a related work [17], a designer cellulosome composed of two *T. fusca* cellulases (endoglucanase Cel5A and exoglucanase Cel48A) converted to the cellulosomal mode was demonstrated to be more efficient in degrading the crystalline cellulose substrate compared to a mixture of the respective wild-type enzymes. The same two cellulases were therefore selected to further compare their performance as bifunctional enzymes.

This report describes a set of bifunctional enzymes produced by gene fusion of catalytic modules together with one or two CBMs. The Cel48A exoglucanase and the Cel5A endoglucanase from *T. fusca* were combined to produce diverse geometrical arrangements, and the resulting chimeras were tested for their activity on microcrystalline cellulose. The results were compared to the parallel action of the two wild-type enzymes and the corresponding designer cellulosome bearing the converted dockerin-containing chimaeras (*b*-48A and *f*-5A).

## Results

### Construction and expression of recombinant proteins

**Figure 1:**
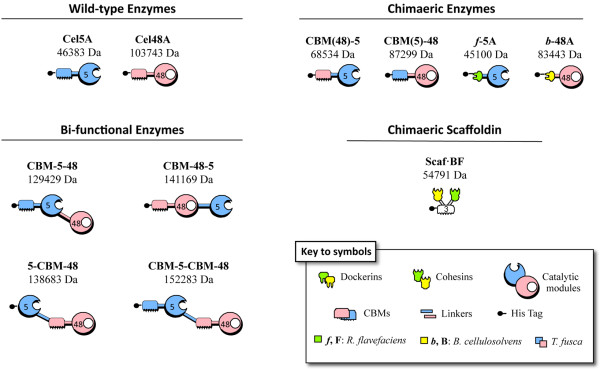
**Schematic representation of the recombinant proteins used in this study.** In the shorthand notation for the engineered enzymes, the numbers 5 and 48 refer to the corresponding GH family (GH5 and GH48) of the catalytic module; upper case characters (B and F) indicate the source of the cohesin module and lower case characters (*b* and *f*) indicate the source of the dockerin module, *B. cellulosolvens* and *R. flavefaciens,* respectively.

The recombinant proteins designed for use in this study are shown schematically in Figure 1. The enzymes used for this work were based on the wild-type *T. fusca* endoglucanase Cel5A and exoglucanase Cel48A. The CBMs of both enzymes are located at the *N*-terminus of the protein in the native state, belong to family 2 and are able to bind microcrystalline and amorphous cellulosic substrates in a similar manner [29-31]. Several modifications of the original wild-type enzymes were designed within the context of the objectives of the study as will be described in the following paragraphs.

First, the CBMs of the two enzymes were interchanged to form the CBM(48)-5 and CBM(5)-48 chimaeras, respectively, in order to explore whether the origin of the CBM influences the enzymatic activities of the cellulases in the free mode.

Next, a series of bifunctional enzyme chimaeras were designed, which contained permutations of both the family 5 and family 48 catalytic module together with the family 2 CBM from the wild-type Cel5A or Cel48A (or both), thus generating the CBM-5-48, CBM-48-5, 5-CBM-48 and CBM-5-CBM-48 bifunctional enzymes (Figure 1).

In addition, the two cellulases were converted to the cellulosomal mode, whereby: (i) the endoglucanase *f*-5A, comprised two fused modules the catalytic module of the family-5 endoglucanase Cel5A from *T. fusca*, and a dockerin at the *N*-terminus from the *R. flavefaciens* ScaA scaffoldin [32], and (ii) the exoglucanase, *b*-48A, comprised the catalytic module of *T. fusca* exoglucanase Cel48A ligated with a dockerin from the *B. cellulosolvens* ScaA scaffoldin, also at the *N-*terminus. The bivalent chimaeric scaffoldin, Scaf·BF was designed to carry two cohesins of divergent specificity, matching those of the latter dockerins, thereby enabling selective incorporation of the two cellulases into a designer cellulosome. The specific modules that comprise the construct are as follows: cohesin 3 from scaffoldin B of *B. cellulosolvens* (designated B), the family 3a CBM from *C. thermocellum*, which binds strongly to cellulose [33], and cohesin 1 from *R. flavefaciens* scaffoldin B (designated F) [32].

### Cohesin-dockerin specificity

The specificity of the cohesins for the chimaeric dockerin-bearing enzymes was examined semi-quantitatively by a sensitive enzyme-linked affinity assay in microtiter plates [34]. Both cohesins in the chimaeric Scaf·BF specifically bound to their respective dockerin and did not bind (or bound very poorly) to other non-matching dockerin-bearing proteins (data not shown). The results were similar to those demonstrated in previous reports [17,18]. The scaffoldin-borne cohesins bound to their matching dockerins just as efficiently as the individual, monovalent (single-cohesin) scaffoldins, indicating that the binding capabilities of the scaffoldin were reliable and selective. All specific cohesin-dockerin interactions were of similar intensity, indicating that similar amounts of protein were bound in each well, supporting a cohesin:dockerin molar equivalent of 1:1.

### Complex formation

Designer cellulosome complex formation was tested by non-denaturing PAGE. Denaturing PAGE was used as a control for verification of sample content. Predetermined stoichiometric mixtures of the enzymes and the scaffoldin resulted in a single band with altered mobility (band strengthened and shifted), indicating that complete or near-complete complexation was achieved in all cases (data not shown). The quality of the assembly of the designer cellulosome components was similar to those of earlier publications [21,35].

### CBM interplay

**Figure 2:**
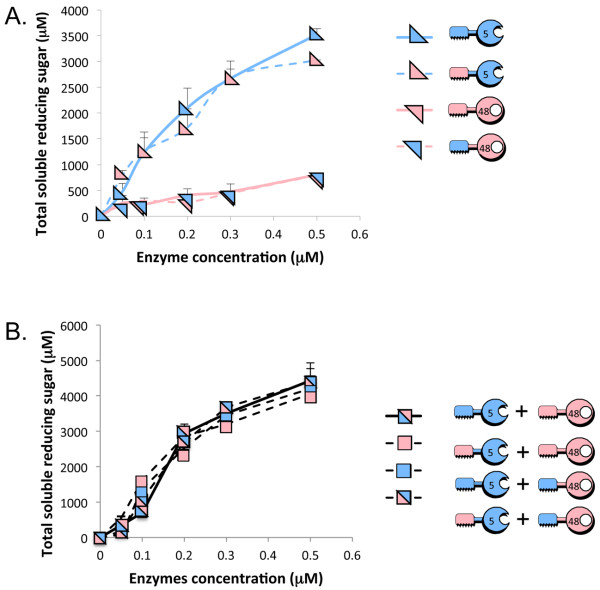
**Influence of CBM source on cellulase activities.A**. Kinetics studies on microcrystalline cellulose hydrolysis by the wild-type cellulases versus the chimaeric cellulases. **B**. Kinetics studies on microcrystalline cellulose hydrolysis by the combination of wild-type versus chimeric cellulases versus mixtures of wild-type and chimaeric enzymes. For convenience, keys and pictograms of the various enzymes are provided. For free enzymes combination, micromolar enzyme concentrations correspond to each one of the enzymes. Triplicates of each reaction were carried out, and standard deviations are indicated.

Interchanging the Cel48A and Cel5A family-2 CBMs did not significantly affect their enzymatic activities on microcrystalline cellulose (Avicel) (Figure 2A). Combinations of the two enzymes, either wild-type, chimaeras or mixture of wild-type and chimaera, exhibited very similar enzymatic activities on microcrystalline cellulose as well (Figure 2B). Thus, the choice of using the family-2 CBM originating from Cel48A or Cel5A during the design of bifunctional enzymes would not be expected to affect their combined activities as bifunctional enzymes. Little or no synergy between the wild-type Cel5A endoglucanase and Cel48A exoglucanase could be observed (Figure 2A and 2B) in line with previous reports [29,36].

### Enzymatic activity of the bifunctional enzymes

**Figure 3:**
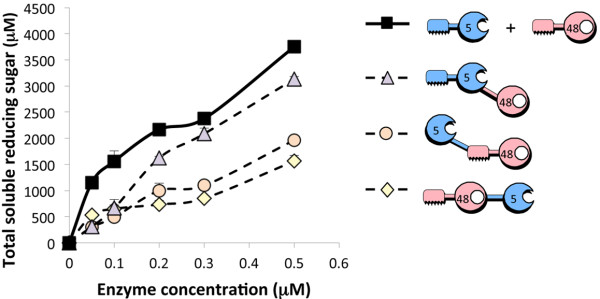
**Kinetics studies on microcrystalline cellulose hydrolysis by the wild-type cellulases versus the bifunctional cellulases.** For convenience, keys and pictograms of the various enzymes are provided. The pictograms denoting the indicated enzymes are defined in Figure 1. For free enzymes combination, micromolar enzyme concentrations correspond to each one of the enzymes. Triplicates of each reaction were carried out, and standard deviations are indicated.

The enzymatic activities on Avicel of the three bifunctional enzymes, CBM-5-48, 5-CBM-48 and CBM-48-5, were compared to the activity of the mixture of the wild-type enzymes Cel48A and Cel5A (Figure 3). The bifunctional enzymes, 5-CBM-48 and CBM-48-5, demonstrated markedly reduced activities relative to the wild-type enzymes (two-fold reduction), whereas CBM-5-48 exhibited only a moderate reduction in activity (~15% reduction at the highest concentrations) towards the wild-type enzyme mixture. These results suggest that within the context of a bifunctional enzyme the position of each module (catalytic module and CBM) has an influence on its overall enzymatic activity.

### Multi-modular enzyme design versus the designer cellulosome approach

**Figure 4:**
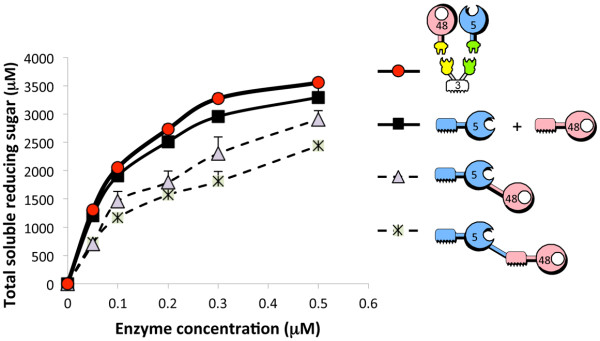
**Kinetics studies on microcrystalline cellulose hydrolysis by the wild-type cellulases versus the bifunctional cellulases and the designer cellulosomes.** For convenience, keys and pictograms (defined in Figure 1) of the various enzymes are provided. For free enzymes combination, micromolar enzyme concentrations correspond to each one of the enzymes. Triplicates of each reaction were carried out, and standard deviations are indicated.

The bifunctional enzyme CBM-5-CBM-48 exhibited a ~20% reduction in enzymatic activity at the highest concentrations on Avicel compared to CBM-5-48 (Figure 4), suggesting that the addition of a second CBM decreased the observed degradation of the microcrystalline cellulosic substrate. The mixture of free wild-type enzymes (shown in Figure 3) was found to be more effective than any of the bifunctional chimaeras, and the designer cellulosome exhibited a minor but statistically significant enhancement (~8%) in enzymatic activity relative to that of the free wild-type enzymes.

## Discussion

In past studies, we have succeeded in “domesticating” the cellulases and xylanases of the well-characterized free enzymatic system of *T. fusca* to work in the cellulosomal mode [16-22]. In the present communication, we examined the conversion of these enzymes into the multifunctional mode, by integrating the catalytic modules of two prominent cellulases of the *T. fusca* system, a family 5 endoglucanase and a family 48 exoglucanase, into a single polypeptide chain. The two cellulases, Cel5A and Cel48A, respectively, have been selected for their known synergistic cooperation on cellulosic substrates [17]. Moreover, both of these GH families occur frequently in bifunctional endo-/exo-type enzymes, despite the fact that combined GH48 and GH5 catalytic modules have yet to be described as collective components of a natural multifunctional enzyme system [1,12,37,38]. Our previous research with these two enzymes [17], however, has demonstrated their compatibility in both the free and cellulosomal modes, which forms solid basis for their combined comparative evaluation in the bifunctional state.

In designing the components of the chimaeric and bifunctional enzymes, careful attention was paid to the precise grafting of the component parts according to predetermined criteria in order to form the final product. In this context, each catalytic module was cloned as a unit together with its intact wild-type linker to which the desired component (CBM, dockerin or enzyme) was appended. When necessary, i.e., for design of 5-CBM-48 and CBM-5-CBM-48, the entire linker of Cel5A was used and inserted at the *C*-terminus of the GH5 catalytic module.

Multifunctional enzymes observed in nature display CBMs belonging to various CBM families including family 2 [1], thus supporting integration of the original family-2 CBMs of the cellulases into the bifunctional chimaeras designed in this work. Interchanging the CBM2 of Cel5A and Cel48A revealed that both CBM2s confer similar levels of enzymatic activities to the catalytic modules either as an intact free enzyme or in combination in the bifunctional state. Therefore, the CBM2 originating from either Cel5A or Cel48A could be used as a viable component in designing a desired bifunctional enzyme without further consideration. The number and position of CBMs in the multifunctional enzyme has an effect on the enzymatic activity of the given chimaera. An inhibitory effect on cellulose degradation was thus observed when two copies of the family-2 CBM were present on the bifunctional enzyme. A similar effect was reported earlier for family 3 CBMs as chimaeric scaffoldin components in designer cellulosomes [14,39]. Nevertheless, the inhibitory effect shown in the present article may not reflect a simple excess of substrate-targeting modules, which may impede the dynamic and concerted action of the two enzymes on the substrate. The data for the bifunctional enzymes shown in Figures 3 and 4 revealed that the position of the various modules on the polypeptide chain is of critical importance to the activity of the enzyme. Thus, it appears that the bifunctional enzyme with the endoglucanase on the *N*-terminus (CBM-5-48) exhibits enhanced cellulolytic activity compared to the *N*-terminally positioned exoglucanase (CBM-48-5). Moreover, the CBM located on the *N-*terminus of the GH5 appears to be critically important to activity, since in its absence the respective chimaera (5-CBM-48) shows markedly reduced levels of cellulose degradation.

The matching set of enzymes and associated components designed for this study allowed us to compare the enzymatic activity on cellulose of the three enzyme paradigms, (i) the wild-type enzymes, (ii) the respective dockerin-containing chimaeras converted to cellulosomal modes and (iii) the enzymes converted to the bifunctional mode. In all cases, the components contain the same GH5 endoglucanase and GH48 exoglucanase. Bifunctional and cellulosomal systems would presumably act with enhanced synergy (in comparison to the wild-type free enzyme system) because the proximity of the enzymes to each other would allow the newly formed free chain ends, created by the endoglucanases, to be exposed to processive action by the exoglucanase. However, in view of our results, only the designer cellulosome paradigm demonstrated minor levels of enhanced enzymatic activity relative to the free enzymes. Possible explanations could be that designer cellulosomes have a higher flexibility that enables more efficient enzymatic activity or that the structural conformations of the catalytic modules upon their fusion affected their enzymatic abilities. In past studies, designer cellulosomes composed of dockerin-bearing converted enzymes from *T. fusca* achieved optimal levels of degradation, which surpassed the activity of the free wild-type enzymes on cellulosic substrates [16-18,20-23].

It appears that designing effective multifunctional enzymes is challenging as the position of the component parts and presumably the length and composition of the linkers will have an impact on the overall activity of the resulting protein. In native multifunctional enzymes, the exact spacing and positioning of the components is the product of lengthy evolutionary selection to optimize synergy between catalytic modules. As a result, the preservation or improvement of the enzymatic characteristics in multifunctional chimaeras may be more difficult than enhancing the enzymatic performance of individual components.

## Conclusions

Although promising fusion proteins have been demonstrated in previous works [26-28], and considerable benefits such as efficient reactivity due to intramolecular synergy, easier optimization of physical charaterization (including pH and temperature) and production of a single protein have been discussed; knowledge of the basic mechanisms is still lacking in this field. In addition, naturally occurring multifunctional enzymes are limited to small numbers of enzymes and their existence as functional components on the same polypeptide chain implies that these enzymes would be expected to work in concert. On the other hand, the presence of two different enzymatic activities on a single polypeptide chain also implies that these activities are confined to equimolar ratios, which are usually sub-optimal [40-44].

Engineering multifunctional enzymes deserves continued research efforts as the formation of multifunctional enzymes could be regarded as the naturally occurring fusion of various complementary sets of catalytic and other modules to perform their respective functions in the hydrolysis of plant cell walls. In addition, both multifunctional enzymes and free enzymes occur together in the certain cellulosome-producing bacterial species, such as *Clostridium thermocellum* or *Ruminococcus flavefaciens*[6,7,10,45-48], thus implying that the cooperative action of the different enzymatic paradigms serves to benefit the overall efficiency of plant cell wall degradation.

## Methods

### Cloning

Wild-type, chimaeric enzymes and recombinant scaffoldins Cel5A, Cel48A, *b*-48A, *f*-5A, and Scaf·BF were cloned as described previously [21,29,30].

Chimaeric enzymes CBM(48)-5, CBM(5)-48, CBM-5-48, 5-CBM-48, CBM-48-5 and CBM-5-CBM-48 plasmids were assembled from catalytic modules and CBM, cloned from *T. fusca* genomic DNA. Family 5 catalytic module was amplified using primers 5^′^-NNNNNNGACGAAGGCTCCGAGCCGGGCGGCCCC-3^′^ and 5^′^-NNNNNNTCAGGACTGGAGCTTGCTCC-3^′^ (where NNNNN represents 4 to 6 random nucleotides that appear before the appropriate restriction site); family 48 catalytic module using 5^′^-NNNNNNCCACCCACCGTCCGCGTGCCGCAGGG-3^′^ and 5^′^-NNNNNNTCAGGGAGCTCCGGCCCCGAACAGT-3^′^; family-2 CBM from Cel5A using 5^′^-NNNNNNGCTGGTCTCACCGCCACAGTCA-3^′^ and 5^′^-GTGCCGGTGCCGGGCTGCGTGC-3^′^ and family-2 CBM from Cel48A using 5^′^-NNNNNNGCCTTCGCCTGCTCGGTGGACTA-3^′^ and 5^′^-NNNNNNTCGATCTCCCGGACCGTCACCT-3^′^. The different modules were assembled in linearized pET28a to form the plasmids.

All enzyme constructs were designed to contain a His-tag for the subsequent purification. PCR reactions were performed using ABgene Reddymix x2 (Advanced Biotechnologies Ltd., United Kingdom) and DNA samples were purified using a HiYield^TM^ Gel/PCR Fragments Extraction Kit (Real Biotech Corporation, RBC, Taiwan).

### Protein expression and purification

Wild-type, chimaeric enzymes and recombinant scaffoldin Cel5A, Cel48A, *b*-48A, *f*-5A, and Scaf·BF were prepared as described previously [21,29,30]. Chimaeric enzymes CBM(48)-5, CBM(5)-48, CBM-5-48, 5-CBM-48, CBM-48-5 and CBM-5-CBM-48 plasmids were expressed in *E. coli* BL21 (lDE3) pLysS cells and purified on a Ni-NTA column (Qiagen), as reported earlier [19]. Scaffoldin, Scaf·BF, was expressed and purified on phosphoric acid swollen cellulose 7.5 mg mL^-1^ pH 7 (PASC) according to the previously described methodology [49]. Purity of the recombinant proteins was tested by SDS-PAGE on 12% acrylamide gels. The concentration of each purified protein was estimated by absorbance (280 nm) based on the known amino acid composition of the protein using the Protparam tool ( http://www.expasy.org/tools/protparam.html). Proteins were stored in 50% (v/v) glycerol at −20°C.

### Affinity-based ELISA

The matching fusion-protein procedure of Barak and coworkers [19,34] was followed to determine cohesin-dockerin specificity.

### Non-denaturing PAGE

To check the full interaction between scaffoldin and enzymes, a differential mobility assay on non-denaturing gels was used. In a 30 μL reaction (in which 15 μL of Tris Buffer Saline pH 7.4 (TBS) buffer, supplemented with 10 mM CaCl_2_ and 0.05% Tween 20), 4 to 8 μg of each protein were added in an equimolar manner. The 1.5 mL tubes were incubated 1.5 h at 37°C. Sample buffer (7.5 μL, in the absence of SDS) was added to 15 μL of the reaction mixture, and the samples were loaded onto non-denaturing gels (4.3%-stacking/9%-separating phase). A parallel SDS-PAGE gel (10%) was performed on the remaining 15 μL sample.

### Enzymatic activity

The enzymes (0 to 0.6 μM) were tested in 200 μL final volume, containing 40 μL microcrystalline cellulose 10% (Avicel, FMC Biopolymer (Philadelphia, PA, USA)), 0.8 μL EDTA (0.5 M), 1.2 μL of CaCl_2_ (2M) and 20 μL of acetate buffer (0.5 M, pH 5). The 1.5 mL tubes were incubated for 18 h at 50°C under shaking conditions, and the reaction was terminated by immersion of the tube in ice water. The samples were then centrifuged for 10 min, at 14,000 rpm and 4°C, 100 μL of the supernatant was added to 150 μL dinitrosalicylic acid reagent (DNS) [50], and the mixtures were boiled for 10 min. The absorbance then was measured at 540 nm. Enzymatic activity was calculated using a glucose standard curve and was expressed in ∞moles of glucose equivalents (reducing sugar) per minute. Dockerin-containing enzymes were subjected to 2 h incubation (37°C, in the absence of substrate) in the presence of equimolar concentrations of scaffoldin, prior to assay for binding interaction. All assays were performed in triplicate.

## Abbreviations

CBM: Carbohydrate-binding module; GH: Glycoside hydrolase; DNS: Dinitrosalicylic acid; PASC: Phosphoric acid swollen cellulose; TBS: Tris buffer saline.

## Competing interests

The authors declare that they have no competing interests.

## Authors’ contributions

SM designed and carried out the experiments, analyzed results and wrote the manuscript. YB, DBW and QX assisted in the experimental design and reviewed the manuscript. MEH and EAB coordinated the study and reviewed the manuscript. All authors read and approved the final manuscript.
